# Clinical phenotype of a Kallmann syndrome patient with IL17RD and CPEB4 variants

**DOI:** 10.3389/fendo.2024.1343977

**Published:** 2024-04-02

**Authors:** Jianmei Zhang, Suhong Yang, Yan Zhang, Fei Liu, Lili Hao, Lianshu Han

**Affiliations:** ^1^ Department of Pediatric Endocrinology and Genetics, Hangzhou Children’s Hospital, Hangzhou, Zhejiang, China; ^2^ Department of Pediatric Endocrinology and Genetic Metabolism, Shanghai Institute for Pediatric Research, Xinhua Hospital, Shanghai Jiaotong University School of Medicine, Shanghai, China

**Keywords:** Kallmann syndrome, delayed pubertal development, gene variant, IL17RD, CPEB4

## Abstract

**Background:**

This study aimed to characterize the clinical phenotype and genetic variations in patients with Kallmann syndrome (KS).

**Methods:**

This study involved the collection and analysis of clinical data from an individual with sporadic KS. Following this, peripheral blood samples were obtained from the patient and his parents. Genomic deoxyribonucleic acid was extracted and subjected to whole-exome sequencing and genomic copy number variation (CNV) detection. Finally, Sanger sequencing was performed to validate the suspected pathogenic variants.

**Results:**

Whole-exome sequencing confirmed that the child carried both the *IL17RD* variant (c.2101G>A, p.Gly701Ser) inherited from the mother and the new *CPEB4* variant (c.1414C>T, p.Arg472*). No pathogenic CNVs were identified in CNV testing.

**Conclusion:**

Bioinformatics analysis shows that the IL17RD protein undergoing Gly701Ser mutation and is speculated to be phosphorylated and modified, thereby disrupting fibroblast growth factor signaling. This study also suggested that the CPEB4 might play a crucial role in the key signaling process affecting olfactory bulb morphogenesis. Overall, the findings of this study broaden the gene expression profile of KS-related pathogenic genes. This offers a new avenue for exploring the pathogenic mechanism of KS and provides valuable insights for precise clinical diagnosis and treatment strategies for this condition.

## 1 Introduction

Idiopathic hypogonadotropic hypogonadism (IHH) is a genetically and clinically heterogeneous disorder, primarily due to insufficient activation of the hypothalamic gonadotropin-releasing hormone (GnRH) axis or failure of pituitary gonadotropin synthesis, secretion, or function (1, 2). During embryonic development, several interacting genes are involved in olfaction establishment and the migration of GnRH neurons, implying a shared embryonic origin among these genes (2–4). Typically, IHH is associated with impaired olfactory function (1, 5), which is a characteristic feature of Kallmann syndrome (KS). KS is characterized by delayed pubertal development, inadequate development of secondary sexual characteristics, and hyposmia or loss of olfactory sensation, present in approximately 60% of IHH cases. IHH cases where olfaction remains normal are referred to as normosmic IHH (nIHH) (1, 6, 7). Furthermore, patients with IHH, influenced by various genes, might also manifest a range of non-reproductive symptoms, such as sensorineural deafness, bimanual synkinesia, dental hypoplasia, renal hypoplasia, skeletal abnormalities, and midline hypoplasia (5, 7, 8).

Over the past few decades, more than 50 genes were associated with IHH, and only some of them were strongly associated, but not the majority. To date, approximately 40% of patients with IHH and approximately 10% of patients with KS have definite genetic defects (9–11), among these, classical KS pathogenic genes include *KAL1、FGFR1、PROKR2* (1, 12–14). *IL17RD* has been confirmed to cause KS, however, few reports have been carried out to understand the contribution of *IL17RD* variants to KS. This case report provides a comprehensive account of the clinical data and diagnostic and therapeutic history of a sporadic KS case attributed to an *IL17RD* variant. Additionally, a review and summary of the characteristics of patients carrying the *IL17RD* variant as reported in the existing literature is offered. Finally, through bioinformatics analysis, the molecular mechanism *via* which the *IL17RD* variant causes KS is uncovered.

## 2 Participants and methods

### 2.1 Research participants

A patient diagnosed with KS in August 2022 was included in this study. The Ethics Committee of our hospital approved this study (approval No.2023-083-01), and informed consent for this clinical research was obtained from the patient’s guardian.

### 2.2 Methods

#### 2.2.1 Clinical data collection

The clinical data of the patients in this study were obtained from the following sources: (1) medical records, encompassing current medical history, past medical history, birth history, and growth and development history; (2) physical examinations involving evaluations of the cranial region, the five sensory organs, and the reproductive system; (3) auxiliary findings, including magnetic resonance imaging (MRI) of the pituitary gland and olfactory bulb, scrotal ultrasonography, digital radiographic (DR) examination of the left wrist joint, and pituitary-related hormone tests.

#### 2.2.2 Whole exon gene sequencing and genomic copy number variation detection

Peripheral blood samples were collected from the patient and his parents, and these samples were processed to extract genomic deoxyribonucleic acid (DNA) after the anticoagulation process. The whole-genome exon region DNA was captured and enriched using sequence capture technology (Agilent SureSelect series kit) and subjected to high-throughput sequencing (Illumina, PE150). The whole-exome gene assay achieved a coverage rate of 99.7% for the intervals with sequencing depth ≥20×. Following sequencing, the obtained data were aligned with reference databases, including the human reference genome and the 1000 Genomes database. Additionally, candidate variants were verified by Sanger sequencing. Parental origin verification of the variants was performed to determine the presence of new-onset variants using the HGMD、ClinVar、OMIM and existing literature. The pathogenicity of these variant loci was assessed according to the American College of Medical Genetics and Genomics standards and guidelines (15).

### 2.3 Bioinformatics analysis

Multiple sequence alignment was performed using Clustal Omega (16). Visualization of amino acid conservation within the mutant proteins was accomplished using Jalview and WebLogo. Pathogenicity predictions for the novel variants were made using MutationTaster and Polyphen (17, 18). Furthermore, the three-dimensional spatial structure of the IL17RD protein was established using AlphaFold online software. InBio Map is used to construct a protein-protein interaction network.

## 3 Results

### 3.1 Clinical data

#### 3.1.1 Medical history

A 14.4-year-old male patient presented with a complaint of “having a short penis for over 7 years and reduced olfaction for over 5 years”. He was born at full term with a birth weight of 3.5 kg and had no history of resuscitation, asphyxia, or exposure to specific medications. By approximately 3 years of age, the patient underwent surgery for cryptorchidism and was diagnosed with allergic rhinitis. His parents had a normal clinical phenotype. His mother conceived naturally after marriage, and there was no history of consanguineous marriage or a family history of hereditary disease.

#### 3.1.2 Physical examination findings

The patient’s physical characteristics included a height of 162 cm, a weight of 58 kg, and a body mass index of 22.1kg/m^2^. His facial appearance was unremarkable, without café au lait spots, laryngeal nodes, axillary hair or beard growth, and any signs of breast development. His pubertal development is in Tanner stage 1, with a penis length of 2 cm and a penis circumference of 1.2 cm. Prader’s testis measurements indicated a left testis volume of 2 mL and a right testis volume of 1 mL. His external genitalia were infantile. Furthermore, his cardiac, pulmonary, abdominal, and neurological examinations did not reveal any abnormalities. During the olfactory test (water, 75% alcohol, vinegar, and perfume), the patient demonstrated an inability to discern smells. Audiometry confirmed sensorineural hearing loss in the right ear.

#### 3.1.3 Laboratory test findings

The patient’s peripheral blood and urine routine tests, liver and kidney function assessments, lipids levels, electrolytes, myocardial enzyme concentrations, alpha-fetoprotein levels, and carcinoembryonic antigen levels were normal. Pituitary-related hormone tests indicated normal adrenocorticotropic hormone (ACTH) and cortisol rhythms and levels, thyroid function, prolactin, and insulin-like growth factor 1. The basal levels of Luteinising hormone (LH), which was 0.22 IU/L (normal reference range 0.71–6.24, the same below), follicle-stimulating hormone (FSH), which was 1.63 IU/L (0.91–7.25), and testosterone (T) was 0.45 nmol/L(0.71–22.92). Additionally, during the gonadorelin stimulation test (1-day method), LH reached a maximum of only 2.71 IU/L at 60 min. The patient’s chromosomal karyotype analysis confirmed a 46, XY pattern.

#### 3.1.4 Imaging findings

**Figure 1:**
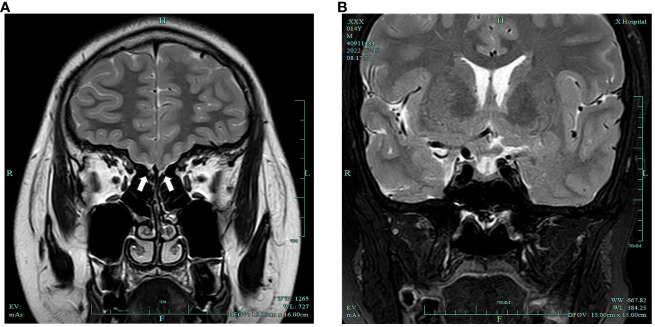
**(A)** Bilateral olfactory nerve deficits (white arrows); **(B)** slightly smaller pituitary gland with no obvious occupying lesions.

DR examination of the left wrist indicated a bone age of 13 years. A pituitary MRI plain scan revealed a small pituitary gland with no significant occupying lesions. Plain MRI imaging of the olfactory bulb indicated bilateral olfactory nerve deficits (**Figure 1**). Scrotal ultrasonography revealed a left testis measuring 1.2cm×0.6cm×0.6cm (volume: 0.23mL) and a right testis measuring 1.0 cm × 0.9 cm × 0.7 cm (volume: 0.33 mL). Ultrasonography of the penis reported a transverse dimension of approximately 1.5 cm × 0.9 cm in the mid-portion, an extracorporeal length of approximately 1.4 cm, and a length from the penis subcutaneous region to the pubic symphysis of approximately 3.3 cm. Moreover, ultrasonography of the liver, gallbladder, pancreas, spleen, thyroid, adrenal glands, both kidneys, and the heart did not reveal any abnormal findings.

### 3.2 Gene sequencing and genomic CNV detection findings

*IL17RD* variant (c.2101G>A, p.Gly701Ser) was detected in the patient. His mother carried the same heterozygous variant, while the father carried the wild-type variant. Importantly, this specific variant was absent in several well-established databases, including OMIM, ClinVar, and LOVD, and lacked any documented polymorphic loci. In addition, the patient also detected *CPEB4* variant (c.1414C>T, p.Arg472*). A comprehensive analysis of the patient’s family via Sanger DNA sequencing confirmed that neither the father nor the mother carried this variant. The *CPEB4* variant was previously unreported in any searches, signifying it as a novel mutation. As for the genomic CNV analysis, no pathogenic or potentially pathogenic CNVs were identified in the patient or his parents.

### 3.3 Online bioinformatics software and protein structure model analysis

**Figure 2:**
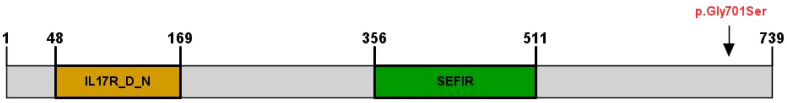
IL17RD protein structural domains and locations of variants observed in this cohort, indicated by arrows (↓).

**Figure 3:**
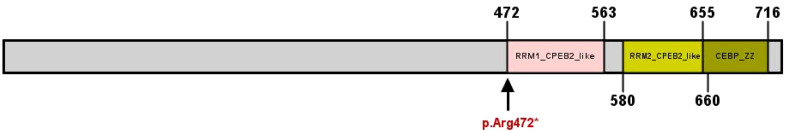
CPEB4 protein structural domains and locations of variants observed in this cohort, indicated by arrows (↓).

**Figure 4:**
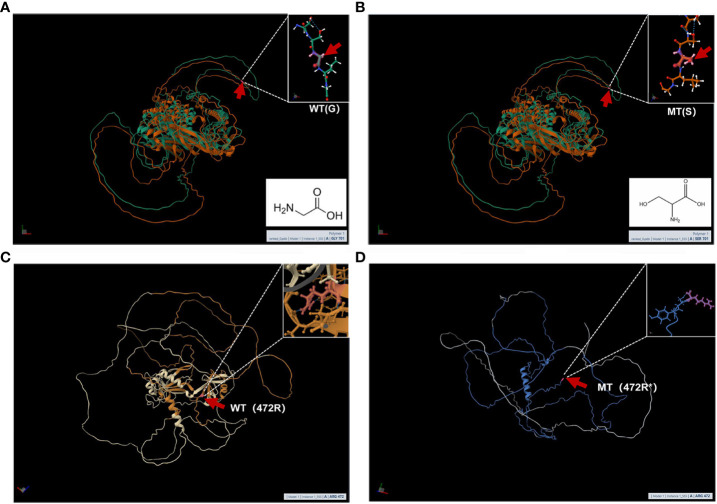
**(A, B)** are the modeling of wild-type and mutant IL17RD proteins respectively: the red arrow represents the mutation position, **(A)** is the wild type and **(B)** is the mutant; **(C, D)** are the modeling of wild-type and mutant CPEB4 proteins respectively: The red arrow represents the mutation position, **(C)** is the wild type, and **(D)** is the mutant.

MutationTaster and Polyphen were employed for pathogenicity prediction of the discovered missense variants, and the results are all harmful. Conservation analysis results indicate that this *IL17RD* variant (p.Gly701Ser) is not located within the conserved domain of the IL17RD protein (**Figure 2**).The *CPEB4* variant (p.Arg472*) is located in a highly conserved region (**Figure 3**). Applying Alpha Fold was used to model the IL17RD protein, and no difference was observed in the structures of the wild-type and mutated *IL17RD* proteins (**Figures 4A, B**).The impact of the variant on the IL17RD protein structure was examined using AlphaFold protein structure prediction. Notably, this variant did not induce substantial alterations in the IL17RD protein’s structure. But surprisingly, it did induce an amino acid change at position #701, substituting glycine with serine. Serine is a recognized site for phosphorylation, implying that this variant could potentially trigger phosphorylation modifications in the protein. We hypothesized whether this variant might cause phosphorylation modification of the protein and thereby regulate the occurrence of the disease. To this end, we used NetPhos software to predict the phosphorylation of IL17RD protein. The results showed that position 701 on the *IL17RD* emerged as a potential phosphorylation site, with a predicted probability value of 0.994 (unsp).Thus, the *IL17RD* protein might undergo phosphorylation modifications after the Gly701Ser mutation. In addition, the *CPEB4* variant(c.1414C>T, p.R472*) was detected, and protein structure predictions were conducted for the CPEB4 protein. This revealed a premature termination of the protein sequence at the arginine residue within the β-fold structure for the wild-type and mutant variants. Specifically, the arginine residue did not form an intact β-fold structure thereafter, thus changing the tertiary structure of the protein and presumably affecting the function of the normal protein (**Figures 4C, D**).

### 3.4 IL17RD and CPEB4 protein interaction network

**Figure 5:**
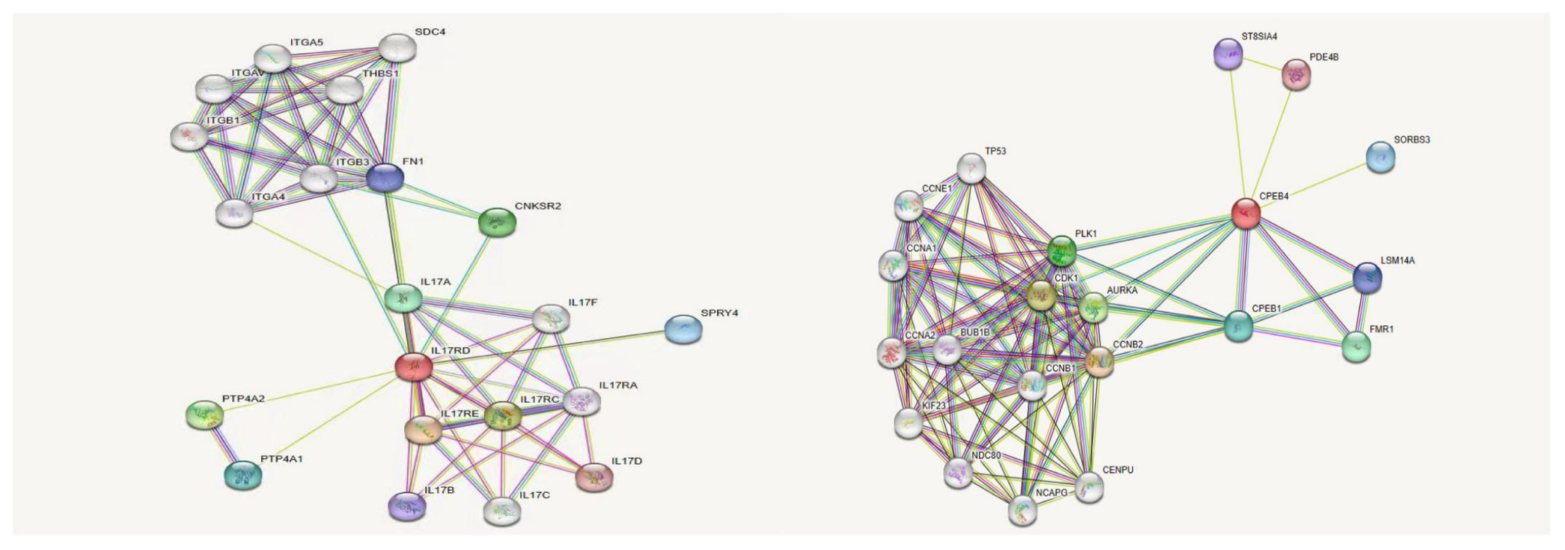
IL17RD and CPEB4 protein interaction network.

We used online bioinformatics software to predict that the *IL17RD* variant (c.2101G>A, p.Gly701Ser) may be the causative mutation of KS. In addition, the *CPEB4* variant was also detected in this patient. Except for the prediction of its possible pathogenicity by using bioinformatics software in this study, no definite evidence related to the pathogenesis of KS could be obtained. Notably, prior research conducted by Tseng et al. has suggested a correlation between the *CPEB4* and olfactory bulb development (19–21). Therefore, there might be an interaction between the CPEB4 protein and the IL17RD protein, contributing to KS pathogenesis. The IL17RD protein interaction network was predicted using InBio Map to verify this hypothesis. Despite not observing a direct interaction between IL17RD and CPEB4 proteins (**Figure 5**), between these two proteins and their synergistic effect on regulating KS pathogenesis warrants further investigation.

### 3.5 Treatment and follow-up

After 4 months of gonadorelin pump pulse therapy (10 µg/90 min), LH levels increased to 4.23 IU/L, FSH increased to 10.69 IU/L, and T increased to 4.79 nmol/L. After treatment, the patient exhibited a left testicular volume of 1.58 mL, a right testicular volume of 2.83 mL, and a penile length of 3.4 cm. The patient has been consistently receiving pituitary pump therapy and has been undergoing regular follow-up examinations for the past year. The most recent examination revealed the following results: T levels at 0.69 nmol/L, LH at 1.71 IU/L, penile length at 3.4 cm, left testicular volume at 2.15 mL, and right testicular volume at 4.37 mL. In addition, adnexal torsion near the right testicle occurred during the follow-up period. Currently, the patient is under ongoing regular follow-up.

## 4 Discussion

Situated on chromosome 3p14.3, the *IL17RD* gene comprises 17 exons. Initially recognized as an inhibitor of fibroblast growth factor (FGF) signaling, IL17RD (also known as Sef) encoded by the *IL17RD* gene, was believed to function as a negative regulator (22, 23). Within the context of this signaling pathway, FGF8 binds to FGFR1, playing a pivotal role in the development and migration of olfactory and GnRH neurons (23, 24). According to a 2013 study by Miraoui et al., IL17RD serves as a signaling hub in the interaction with components of the ERK1/2 mitogen-activated protein kinase cascade and innate immune signaling pathways. IL17RD exerts an antagonistic effect on FGF8-FGFR1 receptor signaling, with dysfunction in this pathway being a key factor in the pathogenesis of IHH (25–27). In the present study, the *IL17RD* variant (c.2101G>A, p.Gly701Ser) was examined for its impact on the IL17RD protein structure using AlphaFold protein structure prediction. However, we speculate that the IL17RD protein can be phosphorylated and modified after the G701S mutation. This process could potentially disrupt FGF signaling, thereby affecting the normal development of GnRH neurons and the olfactory system. It is important to note that this hypothesis warrants further confirmation through large-sample experiments.

Currently, the prevalence of *IL17RD* variants remains unknown, and a definitive correlation between specific *IL17RD* variant types and clinical phenotypes is yet to be established (26, 28, 29). In a study by Zhou et al. (30), which examined mutation profiles and clinical manifestations of 148 Chinese men with IHH, a mutation rate of 3.38% was identified within the *IL17RD*. However, this gene exhibited a significant degree of “incomplete explicitness”. Patients with IHH carrying the *IL17RD* variant from various family lineages displayed varying degrees of hyposmia and hypogonadism. After conducting a literature review, case reports related to KS/IHH induced by this gene variant were summarized after a literature search (**Table 1**). Among them, KS occurred in 18 out of 22 (81.8%) patients, and 72.2% (13 out of 18) of patients experienced other concomitant manifestations of KS, including cryptorchidism, micropenis, hypospadias, hearing loss, abnormal dentition, and osteoporosis. Hearing loss emerged as the most common concomitant symptom (7 out of 18, 38.9%), as confirmed by our findings. In addition, the Gly701Ser mutation carried by the patient in this study has been reported in 2 cases before. One of the KS patients also carried the PROKR2 (p.Y113H) mutation, which has similar clinical manifestations to the patients in this study (30); the other KS patient who carried both FGFR1 (p.Gly348Glu) and RUVBL2 (p.Arg71Trp) only showed non-reproductive symptoms associated with cleft lip and palate (28, 31).

**Table 1:** Currently reported variant sites in the interleukin 17 receptor D gene and corresponding functions.

No.	Sex	Ethnicity	Nucleotide change	Mutation site	Double gene/oligogene mutation	Clinical phenotype	Reference
1	Male	European	c.392A>C	p.Lys131Thr	—	KS, abnormal dentition	(25, 26)
2	Female	European	c.485A>G	p.Lys162Arg	—	KS, hearing loss, osteoporosis	(25)
3	Female	Asian	c.916C>T	p.Pro306Ser	—	KS, hearing loss	(25)
4	Female	European	c.1136A>G	p.Tyr379Cys	*FGFR1* (p.Gly348Arg)	KS, hearing loss, abnormal dentition, osteoporosis	(25)
5	Male	European	c.1403C>T	p.Ser468Leu	—	KS, hearing loss, osteoporosis	(25)
6	Male	Asian	c.1730C>A	p.Pro577Gln	—	KS, hearing loss, abnormal dentition	(25)
7	Male	European	c.2204C>T	p.Ala735Val	*KISS1R* (p.Ala194Asp)	KS, hearing loss	(25)
8	Male	Asian	c.572C > T	Pro191Leu	*—*	nIHH	(17, 25, 27)
9	Male	Asian	c.572C > T	Pro191Leu	*KAL1* (p.Gly552Leu)	nIHH, micropenis	(17)
10	Male	Asian	c.104G> T	Gly35Val	*CHD7 (p.Ser8Arg); CHD7 (p.Lys2129Glu); RNF216 (p.Val249Phe); GNRH1 (p.Gly14Asp); SEMA7A (p.Phe459Ser)*	KS, hearing loss	(27)
11	Female	Asian	c.2012C> T	Ser671Leu	*—*	KS	(27)
12	Male	Asian	c.2012C> T	Ser671Leu	*ANOS1 (p.Trp589X)*	KS, right cryptorchidism	(27)
13	Male	Asian	c.661G> A	Ala221Thr	*FGFR1 (p.Trp190X)*	KS, abnormal dentition, hand deformities	(27)
14	Male	Asian	c.987A> G	Ile329Met	*LEPR (p.1128del); WDR11 (p.Val336Phe); HS6ST1 (p.Pro242Leu)*	KS	(27)
15	Male	Asian	c.985A> G	Ile329Val	*—*	KS, deformity of the external ear	(27)
16	Male	South America	c.878C > T	Pro293Leu	*—*	KS, micropenis, bilateral cryptorchidism	(14)
17	Male	South America	c.2003C>T	p.Ser668Phe	*—*	KS, tetralogy of Fallot, hypospadias, micropenis	(14)
18	Male	South America	c.1697C>T	p.Pro566Leu	*FGFR1 (p.P28L); DMXL2 (p.S1724L)*	nIHH, short stature	(14)
			c.1608_1611del	p.Glu536fs			
19	Male	Asian	c.2101G > A	p.Gly701Ser	*FGFR1(p.Gly348Glu);RUVBL2(p.Arg71Trp)*	KS, cleft lip and palate	(28, 31)
20	Male	Asian	c.1723C>T	p.Arg575Trp	*—*	KS	(29)
21	Male	Asian	c.1592G>T	p.Arg531Met	*—*	KS	(17, 29)
22	Male	Asian	c.2101G > A	p.Gly701Ser	*PROKR2(P.Tyr113His)*	KS	(30)

KS, Kallmann syndrome; nIHH, normosmic isolated hypogonadotropic hypogonadism.

Previous studies have demonstrated that the genetic penetrance and phenotypic expressivity of disease symptoms was dependent on factors such as zygosity of the mutation as well as mutations occurring in other genetic loci, suggesting an oligogenic mode of action (26). IL17RD might also function downstream of gonadotropins to regulate the responsiveness of gonadal tissue and other affected neuronal networks (olfactory and auditory), given that IL17RD is expressed in olfactory placodes in mice (26, 32). Alternatively, given its pattern of oligogenic interactions in disease, IL17RD may epistatically regulate the expression of another gene that may influence these functions (26, 32). Reportedly, cases of KS/IHH caused by *IL17RD* variants had nine out of 21 (42.8%) patients exhibiting double-gene/oligogene inheritance, suggesting that the *IL17RD* often collaborates with other genes in the manifestation of the disease. for instance, in the case of patient #18, who had nIHH without hearing loss, two variants were identified in the *IL17RD* (**Table 1**). These variants included pure missense variants associated with heterozygous frameshifts. Additionally, this patient carried heterozygous variants in the *FGFR1* and *DMXL2*. The *IL17RD* and *FGFR1* variants were categorized as pathogenic, while the *DMXL2* variant was categorized as possibly benign. The contribution of each variant to the disease phenotype remains unknown. To date, *IL17RD* variants have only been reported in patients with KS and nIHH, often exhibiting non-reproductive symptoms such as hearing loss.

Cytoplasmic polyadenylation element binding (CPEB) protein is an RNA binding protein expressed in neuronal tissues including brain and spinal cord that promotes cytoplasmic polyadenylation (33, 34). Vertebrates contain four members of the CPEB family of proteins that are designated CPEB1-4 (19, 20). CPEB1, the founding member of this family, has become an important model for illustrating general principles of translational control by cytoplasmic polyadenylation in gametogenesis, cancer etiology, synaptic plasticity, learning, and memory (35). In studies observing *CPEB* knockout mice, it was found that CPEB1 plays an important role in mouse gametogenesis (35). It was found that the germ cells from the knockout mice harbored fragmented chromatin, suggesting a possible defect in homologous chromosome adhesion or synapsis (21, 33, 36). CPEB1 has essential functions in mouse gametogenesis. Both male and female CPEB1 knockout mice are sterile (33). Although CPEB4 is more similar to CPEB2 and CPEB3 at the amino acid sequence level, previous studies have shown that CPEB4 is more functionally related to CPEB1 and can in turn promote polyadenylation-induced oocyte RNA translation (36). In this study, the *CPEB4* variant (c.1414C>T, p.Arg472*) was detected in this child, resulting in a position change to 472 after translation termination. The alteration disrupts all structural domains and consequently impairs the complete β-folding of the CPEB4 protein, thereby affecting the protein’s structural stability. Bioinformatics revealed that *CPEB4* variant might possess pathogenic potential. However, concrete evidence linking *CPEB4* variants to KS/IHH or other genetic diseases is currently lacking. In summary, we speculate that changes in CPEB4 protein function caused by *CPEB4* variants are likely to affect CPEB1 protein function and thereby disrupt germ cell differentiation, but this needs to be confirmed in future studies.

The mammalian olfactory bulb (OB) is the first relay station for olfactory perception and require continuous replenishment of interneurons (mainly granule cells [GCs]) to support local circuits throughout life (19, 37). Ching-San Tseng et al. found that in the first 2 postnatal weeks in mice, CPEB4 acts as a survival factor exclusively for early postnatal GCs, and olfactory experience initiates CPEB4-activated c-Fos mRNA translation (21). In *CPEB4* knockout mice, c-FOS insufficiency reduced neurotrophic signaling to impair GC survival and cause OB hypoplasia (20, 21). Therefore, the *CPEB4* might be involved in key signaling processes that affect olfactory bulb morphogenesis. The children in this study had hyposmia, which may be related to changes in protein function caused by mutations in the *CPEB4*. At present, it remains unclear whether there is a correlation between CPEB4 and KS and may indicate a novel double-gene pathogenesis of KS/IHH. Further investigation is required to explore the interactions between the genes and proteins involved in the KS/IHH pathway to optimize *in vitro* functional experiments.

## 5 Conclusion

In summary, since there is currently unclear molecular mechanism for most KS patients, further research is needed. On this basis, this study provides convincing evidence to elucidate the molecular mechanism of *IL17RD* variant, and speculates that *CPEB4* may be a new causative gene for KS/IHH, providing a scientific basis for precise clinical diagnosis and treatment.

## Ethics statement

This study was conducted in accordance with the principles of the Declaration of Helsinki. The Ethics Committee of Hangzhou Children's Hospital approved this study. Written informed consent for publication of the participant's clinical details was obtained from the parent.

## Author contributions

ZJ: Writing – original draft, Data curation. YS: Data curation, Supervision, Writing – review & editing. ZY: Data curation, Writing – review & editing. LF: Data curation, Investigation, Writing – review & editing. HaoL: Data curation, Supervision, Writing – review & editing. HanL: Conceptualization, Writing – review & editing.
